# Genome-Wide Identification Analysis of GST Gene Family in Wild Blueberry *Vaccinium duclouxii* and Their Impact on Anthocyanin Accumulation

**DOI:** 10.3390/plants13111497

**Published:** 2024-05-29

**Authors:** Wei Lv, Liyong Zhu, Lifa Tan, Lei Gu, Hongcheng Wang, Xuye Du, Bin Zhu, Tuo Zeng, Caiyun Wang

**Affiliations:** 1 School of Life Sciences, Guizhou Normal University, Guiyang 550025, China; lvwei@gznu.edu.cn (W.L.); 211109040020@gznu.edu.cn (L.T.); leigu1216@nwafu.edu.cn (L.G.); wanghc@gznu.edu.cn (H.W.); duxuye@gznu.edu.cn (X.D.); 201703008@gznu.edu.cn (B.Z.); 2 National Key Laboratory for Germplasm Innovation & Utilization of Horticultural Crops, College of Horticulture & Forestry Sciences, Huazhong Agricultural University, Wuhan 430070, China; zly123456@webmail.hzau.edu.cn

**Keywords:** wild blueberry, *GST* genes, *Vaccinium duclouxii*, anthocyanins

## Abstract

*Vaccinium duclouxii*, a wild blueberry species native to the mountainous regions of southwestern China, is notable for its exceptionally high anthocyanin content, surpassing that of many cultivated varieties and offering significant research potential. Glutathione S-transferases (GSTs) are versatile enzymes crucial for anthocyanin transport in plants. Yet, the GST gene family had not been previously identified in *V. duclouxii*. This study utilized a genome-wide approach to identify and characterize the GST gene family in *V. duclouxii*, revealing 88 GST genes grouped into seven distinct subfamilies. This number is significantly higher than that found in closely related species, with these genes distributed across 12 chromosomes and exhibiting gene clustering. A total of 46 members are classified as tandem duplicates. The gene structure of *VdGST* is relatively conserved among related species, showing closer phylogenetic relations to *V. bracteatum* and evidence of purifying selection. Transcriptomic analysis and qRT-PCR indicated that *VdGSTU22* and *VdGSTU38* were highly expressed in flowers, *VdGSTU29* in leaves, and *VdGSTF11* showed significant expression in ripe and fully mature fruits, paralleling trends seen with anthocyanin accumulation. Subcellular localization identified *VdGSTF11* primarily in the plasma membrane, suggesting a potential role in anthocyanin accumulation in *V. duclouxii* fruits. This study provides a foundational basis for further molecular-level functional analysis of the transport and accumulation of anthocyanins in *V. duclouxii*, enhancing our understanding of the molecular mechanisms underlying anthocyanin metabolism in this valuable species.

## 1. Introduction

The genus *Vaccinium* is renowned for its high-quality berry fruits, notably blueberries, distinguished by their substantial content of bioactive compounds such as anthocyanins, flavonoids, and phenolic acids [1]. For example, the highbush blueberry (*Vaccinium corymbosum*), a commercially significant variety, contains anthocyanins that constitute 60% of its total polyphenolic content [2]. In comparison, *Vaccinium myrtillus* boasts an anthocyanin concentration accounting for 90% of the total phenolic compounds in the fruit [3,4]. These levels far exceed those found in many other fruits. In blueberries, the predominant anthocyanidins include cyanidin, delphinidin, petunidin, peonidin, and malvidin [5]. Compared to other nutritional components in fruits, blueberries possess a notably higher anthocyanin content, with a total of 5.583 mg/g, surpassing other berries such as raspberries, cherries, and strawberries [6]. High-performance liquid chromatography-tandem mass spectrometry (HPLC-MS/MS) analysis reveals that the total phenolic content in blueberries is significantly higher than strawberries [7].

Anthocyanins, naturally occurring pigments with chemical structures that include catechol, pyrogallol, and methoxy groups, not only serve as natural colorants in blueberries but also demonstrate significant antioxidant, antimicrobial, anti-apoptotic, and anti-inflammatory activities. These properties make anthocyanins a major focus in both pharmaceutical and food industries [8,9]. These bioactive compounds are potent anti-inflammatory agents and play a critical role in preventing and managing chronic conditions associated with oxidative stress, such as cardiovascular diseases and diabetes [10,11]. Furthermore, the consumption of berry juices rich in anthocyanins can boost human immunity and enhance overall health and wellness. Additionally, blueberry-derived anthocyanins have a significant prebiotic effect, positively impacting the gut microbiome and enhancing gut health [12]. Beyond their consumption as raw fruits, blueberries’ versatility extends to various products, including juices, wine, vinegar, jams, dried fruits, and fruit pulp powders. They are also utilized as colorants and flavor additives in a wide range of food products, from baked goods like cakes, cookies, and bread to dairy products like yogurt and jellies, demonstrating their practical and economic value [1,13,14]. Consequently, enhancing the anthocyanin content in blueberries has become one of the focal points in breeding programs aimed at developing varieties with greater nutritional and commercial value.

Glutathione S-transferases (GSTs, EC 2.5.1.18) play a pivotal role in the plant kingdom. As part of a large and ancient protein superfamily, GSTs are found almost universally across all organisms and exhibit functional diversity in plants [15]. They are involved in various processes, including growth and development, plant hormone signal transduction, regulation of redox states, and biosynthesis. Specifically, GSTs are crucial for the sequestration and transportation of anthocyanin secondary metabolites into vacuoles, acting as vital non-catalytic carrier proteins. Early research revealed that the intracellular transport of anthocyanins involves GST-mediated processes, alongside membrane transport and vesicular trafficking, highlighting their integral role in moving these pigments from the cytoplasm into vacuoles [16,17,18]. High expression levels of GSTs are conducive to the enhanced transfer and accumulation of anthocyanins. For instance, in *Malus domestica*, the expression of *MdGSTU12* is positively correlated with anthocyanin content and genes involved in their synthesis [19]. In *Actinidia chinnensis*, *AcGST1* is localized in the endoplasmic reticulum and vacuolar membrane, with its expression levels closely aligned with anthocyanin accumulation, suggesting its role as a carrier protein [20]. Additionally, overexpression of *StGST*1 in *Solanum tuberosum* promotes anthocyanin accumulation in tubers [21]. In *Prunus persica*, the gene *Pp3G013600* encodes an anthocyanin-transporting GST, where genetic variants such as a 2bp insertion or a 5bp deletion in the third exon could potentially disrupt function, affecting anthocyanin presence in fruit epicarp [22].

*Vaccinium duclouxii*, a perennial small wild berry fruit tree from the Ericaceae family, is predominantly found and endemic to the southwest region of China. It exhibits higher and more comprehensive nutritional components compared to cultivated varieties, marking it as a significant source of valuable traits [23]. This species also demonstrates strong environmental adaptability and stress resistance [24]. Notably, it contains anthocyanins throughout the epicarp and the pulp, unlike cultivated blueberries, making it a species with considerable research potential. Our research team has conducted *Telomere-to-Telomere* (*T2T*) genome sequencing of *V. duclouxii*, producing the highest quality genome data thus far for the *Vaccinium* genus [25]. This achievement provides a robust foundation for exploring desirable traits in *Vaccinium* species. Building upon the existing genome data from other *Vaccinium* species, we are now poised to investigate the functions of the GST gene family and its role in the accumulation of anthocyanins in *V. duclouxii*. This research lays a foundation for future efforts to develop high-quality new blueberry cultivars, promising advancements in agricultural practices and commercial berry production.

## 2. Result

### 2.1. Identification Bioinformatics, and Phylogenetic Analysis of the VdGSTs Gene Family

Conservation and domain analyses were performed on the proteins using the T2T complete genome protein sequences of *V. duclouxii*. Comparisons with the *Arabidopsis thaliana* GST gene family were conducted using Blastp, and the results were merged and de-duplicated with those from Hmmer3.0. Sequences lacking conserved domains were manually excluded. Candidate proteins were validated through the Conserved Domain Database (CDD). Ultimately, 88 *VdGSTs* were identified and characterized. A similar approach identified 54 GST gene family members in *V. bracteatum*. The 88 GST genes were renamed according to their chromosomal positions and familial classifications, and the molecular characteristics of the protein sequences were analyzed (Table S1). The 54 *VbGST* genes were named using the same methodology.

**Figure 1:**
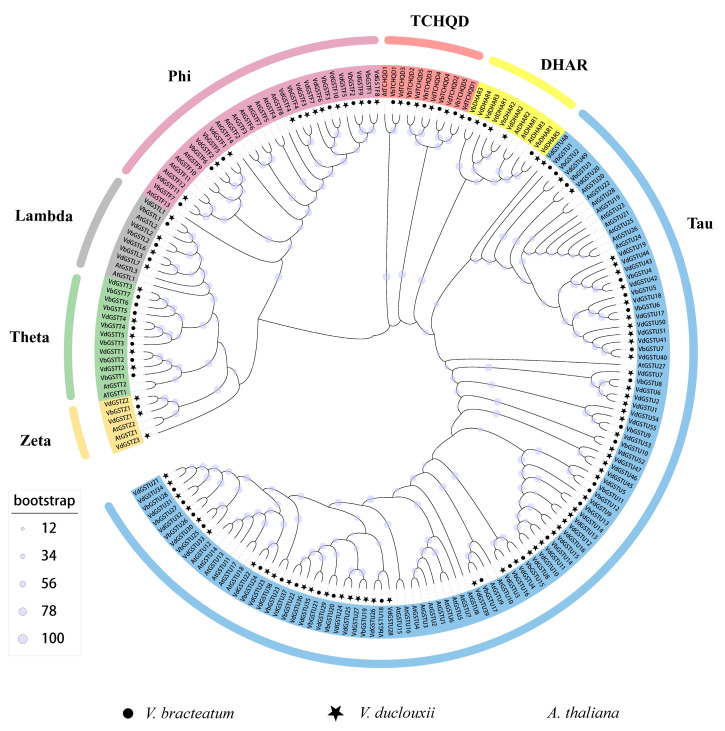
Phylogenetic tree of *V. duclouxii*, *A. thaliana*, and *V. bracteatum* GST gene families. This unrooted phylogenetic tree was constructed using the maximum likelihood (ML) method with a bootstrap repetition value of 1000 times. The tree categorizes the 88 GST genes from *V. duclouxii*, 52 GST genes from *A. thaliana*, and 54 GST genes from *V. bracteatum* into seven subclasses.

The amino acid sequences of 52 AtGSTs from *A. thaliana*, 88 VdGSTs from *V. duclouxii*, and 54 VbGSTs from *Vaccinium bracteatum* were aligned using the MAFFT software v7.158. Following sequence alignment, the phylogenetic tree was constructed using the iqtree2.0 software, employing the maximum likelihood method and bootstrap with 1000 replicates. The tree’s branches and conserved domains indicated that the *VdGSTs* can be classified into seven subclasses: Tau, Phi, Lambda, Zeta, DHAR, TCHQD, and Theta (Figure 1). Consistent with most plant GST families, the Tau subclass is the most populous with 55 members; followed by the Phi subclass with 11 members; and the Theta, TCHQD, and DHAR subclass, each with 5 members. The Lambda subclass comprises four members, while the Zeta subclass is the smallest, with only three members. In *V. bracteatum*, there are 27 Tau members; 11 Phi members; and 5 members each in Theta, DHAR, and TCHQD subclasses, with 4 in Lambda and 3 in Zeta. The Phi subclass, which is mainly related to anthocyanin transport and synthesis, shows a greater number of members in *V. duclouxii* (seven members) compared to *V. bracteatum*.

Physicochemical properties analysis of the 88 VdGSTs revealed that their amino acid sequence lengths varied from 69 (VdGSTL3) to 718 (VdGSTU13) residues, with molecular weights ranging from 7860.86 Da (VdGSTL3) to 81,295.27 Da (VdGSTU13). The isoelectric points (pI) ranged from 4.38 (VdGSTL3) to 9.23 (VdGSTT5), with 12 of the VdGSTs having a pI above 7, indicating a basic nature, while the remaining 76 were acidic. The instability index of these proteins ranged from 20.94 (VdDHAR1) to 53.92 (VdGSTU52), with 35 of them being categorized as unstable (index > 40). Based on the Grand Average of Hydropathy (GRAVY), 10 proteins, including VdGSTU1, VdGSTU5, VdGSTU6, VdGSTU17, VdGSTU22, VdGSTU35, VdDHAR1, VdDHAR2, VdDHAR5, and VdTCHQD3, showed positive hydropathy indices, indicating hydrophilicity. The remaining 78 proteins had negative values. VdDHAR1 had the highest hydropathy index at 0.367, while VdTCHQD5 had the lowest at −0.702. Subcellular localization results indicated that most VdGSTs were located in the cytoplasm, with a few localized to the nucleus and chloroplasts.

### 2.2. Chromosomal Localization and Synteny Analysis of the VdGSTs Gene Family

**Figure 2:**
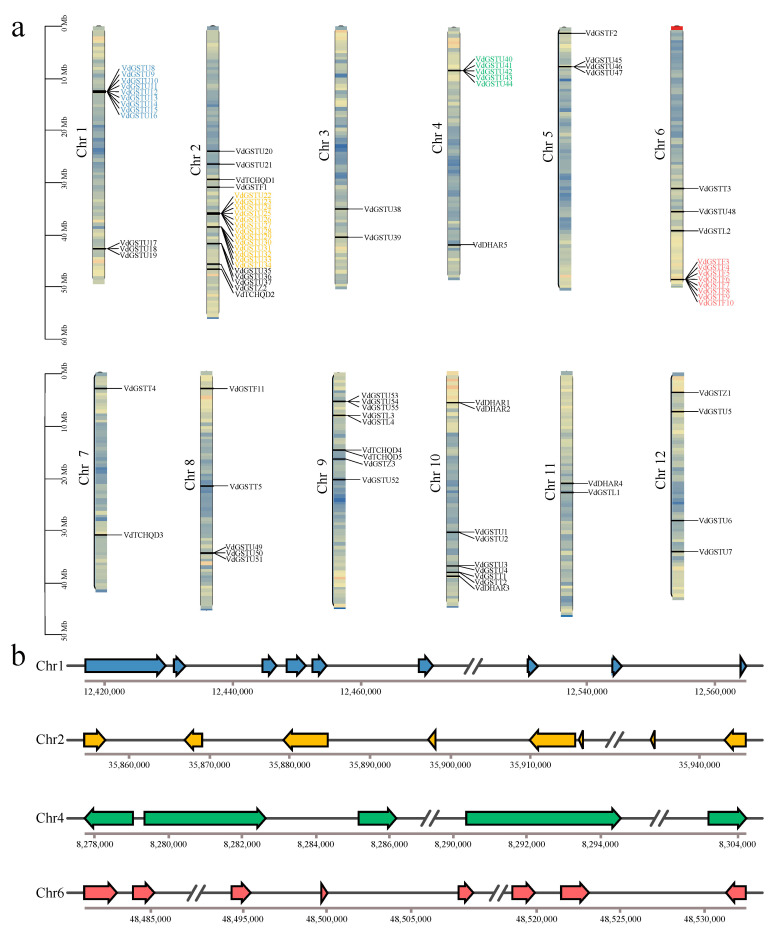
Chromosomal distribution and location of *VdGSTs*. (**a**) Chromosomal localization of *VdGSTs*; (**b**) structural diagram of *VdGSTs* gene clusters.

Based on genome annotation data, the chromosomal localization analysis of the 88 VdGSTs genes shows that they are unevenly distributed across 12 chromosomes (Figure 2a). Chromosome 2 harbors the most members with 21 *GST* genes, while chromosome 7 has the fewest with only 2. Notable gene clusters are present, and the gene cluster structures on chromosomes 1, 2, 4, and 6 (Figure 2b).

**Figure 3:**
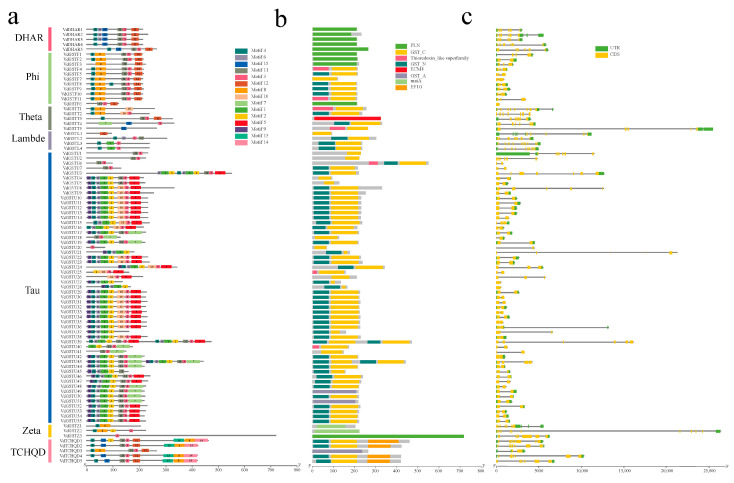
Motifs and DNA structures of the VdGSTs. (**a**) Conserved motif; (**b**) conserved domain; (**c**) gene structure. The scale bar indicates gene length (bp) or protein sequence length, and different colored boxes indicate patterns.

The MEME software (http://meme-suite.org/ accessed on 10 December 2023) was used to analyze 15 conserved motifs (Figure 3a). Motifs 1, 3, and 4 have been identified as conserved among VdGSTs and are traceable across almost all VdGST subfamilies. There are distinct differences in motif composition between different subfamilies. The TCHQD subfamily contains motifs 4, 15, 11, 3, 12, 13, 8, and 14, indicating a unique evolutionary path for this subfamily. The Phi subfamily primarily comprises motifs 4, 8, 11, 3, and 12. In contrast, the DHAR subfamily includes motifs 4, 6, 15, 11, 3, and 12, differing from the Phi subfamily only by the absence of motif 8. Unique motifs 13 and 14 in the TCHQD subfamily suggest its distinct evolutionary trajectory. In the study of conserved domains within the VdGSTs, it is noted that genes in the Tau subfamily almost invariably contain the standard GST-C and GST-N domains (Figure 3b).

The exon and intron structures of the 88 GST genes were visualized using TBtools (Figure 3c) [26]. Results indicate that GST family members within the same subclass have almost identical conserved structural domains, yet significant differences exist between subclasses. The number of introns in GST genes ranges from 1 to 8, while the number of exons varies from 0 to 7. Most genes in the Tau subfamily contain two exons, whereas all members of the Phi subfamily consist of three exons. The Lambda subfamily shows the highest number of six exons. The acquisition or loss of introns can alter gene structure and play a crucial role in the evolution of gene families [27]. These findings suggest that the exon-intron structure of genes within the same category is relatively conserved and closely linked to the evolution of the GST family.

### 2.3. Duplication, Synthesis, and Evolutionary Analysis of VdGSTs Genes Family

**Figure 4:**
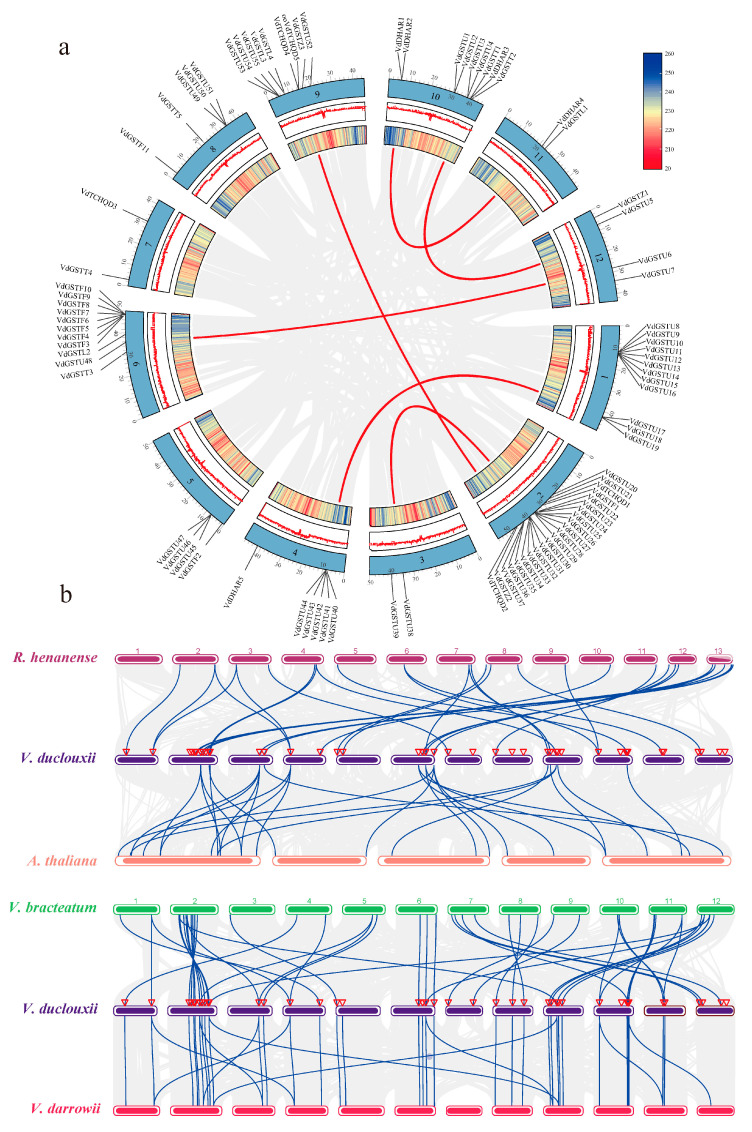
Co-linearity analysis of the *VdGSTs* gene family. (**a**) Co-linearity analysis of the *VdGSTs* gene family in *V. duclouxii*. The red lines indicate segmental duplicate gene pairs of *VdGSTs*, while the gray lines indicate the covariance blocks in the *V. duclouxii* genome. The first circle shows gene density, and the second displays genomic GC content; (**b**) co-linearity analysis of the GST genes of *V. duclouxii*, *V. bracteatum*, *V. darrowii*, *R. henanense*, and *A. thaliana*. The red lines indicate the segmental duplicate gene pairs of *V. duclouxii* GST genes with other species, while the gray lines depict the covariance gene blocks between *V. duclouxii* and other genomes. The numbers above the color blocks represent the chromosomal naming order.

We used the Multiple Collinearity Scan toolkit (MCScanX) to analyze duplication events in GST genes, visualized using TBtools (Figure 4a). Among the 88 GST genes, 46 members were classified as tandem duplications, 11 as whole genome duplications (WGDs) or segmental duplications, 19 as dispersed duplications, 11 as proximal duplications, and one single duplication, *VdTCHQD3* (Table S2). This analysis identified six pairs of duplicated genes within the species.

To further elucidate the *VdGSTs* gene family’s potential evolutionary mechanisms, we analyzed gene duplication events using the MCScanX toolkit. We selected homologous genes from *V. duclouxii*, *A. thaliana*, *R. henanense*, *V. darrowii*, and *V. bracteatum* (Figure 4b). A total of 28 pairs of homologous genes were identified between *V. duclouxii* and *A. thaliana* and 33 pairs between *V. duclouxii* and *R. henanense*. There were 42 homologous genes between *V. duclouxii* and *V. darrowii* and 48 pairs between *V. duclouxii* and *V. bracteatum*. The interactions with *V. darrowii* and *V. bracteatum* involved 37 and 40 *VdGSTs* genes, respectively, more than those with *A. thaliana* and *R. henanense*, which had 17 and 30 genes, respectively. This difference may be attributed to the closer phylogenetic proximity of *V. duclouxii* with these species, especially with *V. bracteatum*.

### 2.4. Analysis of Non-Synonymous (Ka) and Synonymous (Ks) Substitution Patterns in Protein-Coding Genes

**Figure 5:**
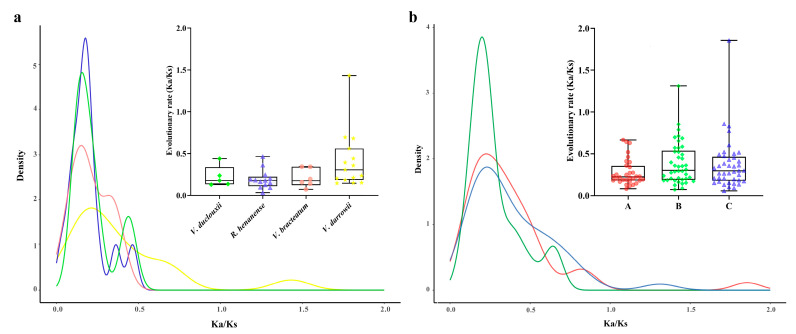
Ka/Ks values of *GST* gene families of four genera. (**a**) Ka/Ks values of GST gene families within the species of *V. duclouxii*, *R. henanense*, *V. bracteatum*, and *V. darrowii*; (**b**) inter-species Ka/Ks values of GST gene families between the species. A: *V. duclouxii*–*R. henanense*; B: *V. duclouxii*–*V. bracteatum*; and C: *V. duclouxii*–*V. darrowii*.

The patterns of Ka and synonymous Ks nucleotide substitutions are critical indicators of gene evolution. The Ka/Ks ratio is used in genetics to determine whether protein-coding genes are under selective pressure and to assess their divergence rates. A Ka/Ks ratio greater than 1 indicates positive selection, a ratio of 1 indicates neutral selection, and a ratio less than 1 suggests purifying selection. This study calculated the Ka/Ks ratios for GST gene families within four species of the genus *Vaccinium* to explore if the GST genes are associated with selective pressure following duplication events. Genes with either a Ka or Ks value of zero were excluded, resulting in intraspecific Ka/Ks values for the four species GST gene families (Figure 5a). The average Ka/Ks ratio was 0.265. Within *V. darrowii*, one pair of genes exhibited a Ka/Ks ratio of 1.432, indicative of positive selection; all other gene ratios were under purifying selection, ranging from 0.032 to 0.791, with most between 0.105 and 0.196. Similarly, interspecific Ka/Ks ratios were calculated for the GST gene families among the four related species, excluding genes with a Ka or Ks value of zero (Figure 5b). These GST genes experienced strong purifying selection within and between the species of the *Vaccinium* genus.

### 2.5. Identification of Cis-Acting Regulatory Elements in the Promoter Regions of the VdGSTs

**Figure 6:**
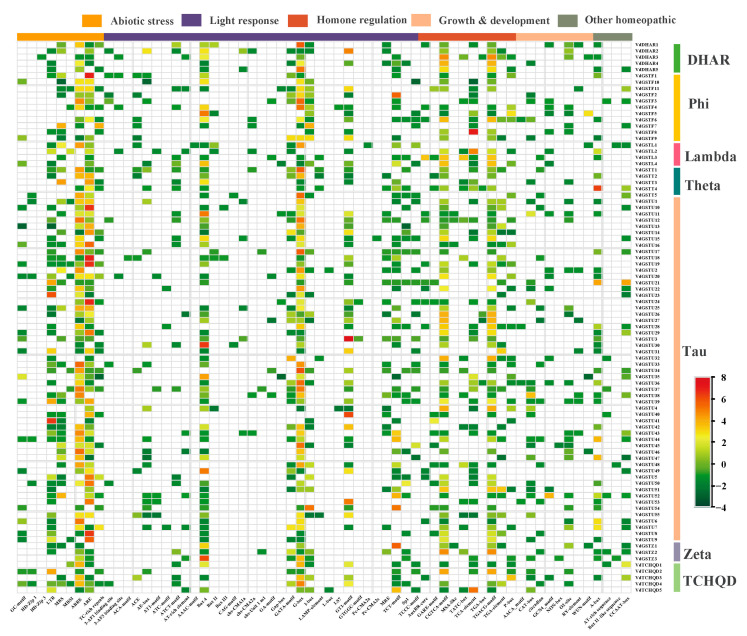
Predictive heatmap of cis-acting elements in the promoters of the *VdGSTs* gene family. The heatmap represents normalized cis-acting counts of elements on promoters.

Using the *T2T* genome sequence and GFF files of *V. duclouxii*, the promoter regions of the *VdGSTs* genes were analyzed via the PlantCARE online platform to predict cis-acting regulatory elements. These elements include ABRE, Box 4, CAT Box, CGTCA motif, GT1 motif, GATA motif, MRE, and MBS, which can bind to transcription factors and regulate various biological processes in plants. Functionally, these cis-acting elements can respond to biotic and abiotic stresses, plant hormones, light, drought, and cold (Figure 6), suggesting the potential multifunctionality of GST genes. Additionally, binding sites for MYB transcription factors, which are associated with anthocyanin synthesis, were also identified within these promoters.

### 2.6. Transcriptome Data Analysis and Expression Analysis of VdGSTs Genes in Various Tissues

**Figure 7:**
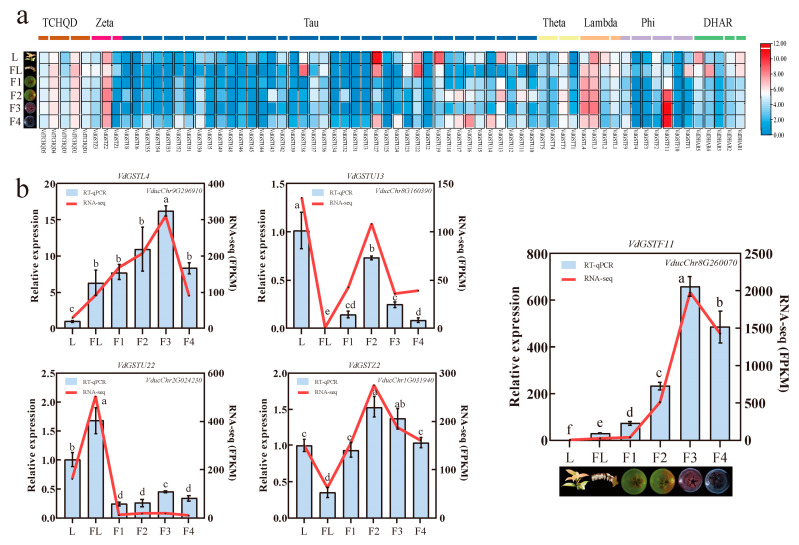
The expression patterns of the GST gene family in the leaves, flowers, and fruits of *V. duclouxii*. (**a**) Relative expression profiles of 68 *VdGSTs* gene family. L, leaves; FL, flowers; F1, green fruits; F2, half-red fruits; F3, red fruits; F4, whole black fruits. The color scale on the right indicates the log_2_ (FPKM + 1) values; (**b**) RNA-seq gene validation using qPCR. Lower-case letters indicate a significant difference (*p* < 0.05) from one-way ANOVA followed by a post-hoc Tukey test.

RNA-seq data show the relative expression of the *VdGSTs* gene family across six tissues: leaves (L), flowers (FL), green fruits (F1), half-red fruits (F2), red fruits (F3), and whole black fruits (F4). Genes with average FPKM values below 1 were excluded. A heatmap displays the expression levels of 68 VdGSTs genes in *V. duclouxii* during different stages of fruit development, from green to mature fruits, and also in leaves and flowers (Figure 7a). The results highlight differential expression across these tissues, with the Phi subfamily gene *VdGSTF11* showing high expression levels during the F2, F3, and F4 stages of fruit development, suggesting its likely involvement in the transfer of anthocyanins. This aligns with reports that the Phi subfamily primarily participates in anthocyanin deposition and transfer. *VdGSTU22* and *VdGSTU38* show high expression in flowers, and *VdGSTU29* is significantly expressed in tender leaves, all belonging to the Tau subfamily (Figure 7b).

### 2.7. Validation of VdGSTF11 Expression by qRT-PCR and Its Subcellular Localization

**Figure 8:**
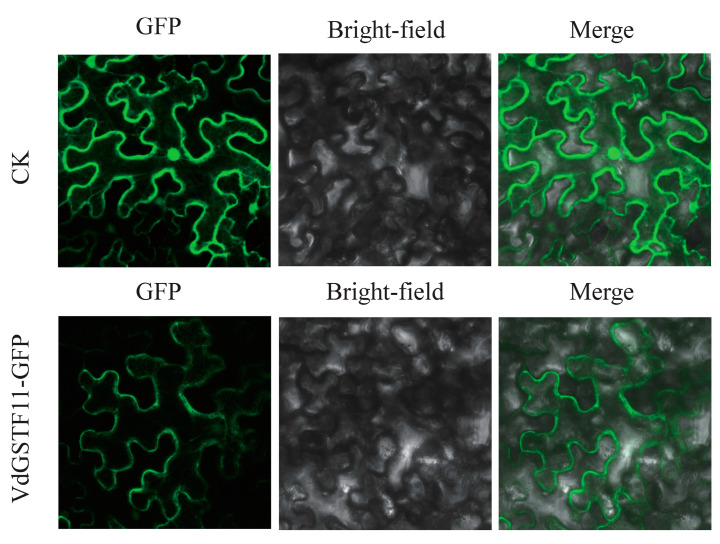
Subcellular localization of *VdGSTF11*.

To further validate the gene expression profiles obtained from RNA-Seq and the reliability of the assembled transcriptome, qRT-PCR analysis was conducted on five key enzyme genes, specifically *VdGSTU19*, *VdGSTU22*, *VdGSTU27*, *VdGSTF11*, and *VdGSTL3*. The result showed that the RNA-Seq and qRT-PCR expression trends were generally consistent. To determine the subcellular localization of *VdGSTF11*, its coding sequence was fused with green fluorescent protein GFP (Figure 8). When transiently expressed in tobacco leaves, distinct fluorescence was observed in the cell membrane, indicating that *VdGSTF11* is localized at the cell membrane and likely plays a key role in anthocyanin transport.

## 3. Discussion

GSTs have been shown to play crucial roles in the accumulation of anthocyanins. In the tree peony (*Paeonia suffruticosa*), 54 *GST* genes were identified, with *PsGSTF3* potentially involved in the transport of petal anthocyanins [28]. In *Raphanus sativus*, 82 *GST* genes were detected, with transcripts of *RsGSTF12-1* and *RsGSTF12-2* being candidate genes encoding anthocyanin transport proteins in radish varieties [29]. In *Prunus persica*, 54 *GST* gene family members were identified, with *PpGST1* confirmed to be responsible for anthocyanin transport and co-regulating anthocyanin accumulation with *PpMYB10.1* [30]. Viral-induced gene silencing of the Phi subfamily member *PsGSTF3* reduced anthocyanin accumulation and the expression of structural genes within the anthocyanin biosynthetic pathway [31]. *VviGSTs* exhibit distinct specificity for flavonoid ligands in *Vitis vinifera*, with *VviGST4* expressed highly in the pericarp during fruit color transition phases, closely associated with anthocyanin accumulation. Experiments also revealed that GST-mediated flavonoid transport involves glutathione dependency [32].

*V. duclouxii* is a species of the blueberry genus predominantly found in the Ailao Mountains of Yunnan province China, thriving at elevations between 1550 and 2600 m. These plants can reach heights from 1 to 5 m, and in Yunnan, ancient trees over 10 meters tall have been documented [25]. The species exhibits several advantageous traits in its competitive local environment. It is tolerant of infertile soil and resilient to adverse conditions, with a deep root system that enables it to prosper on poor lands. Notably, its anthocyanin content significantly surpasses commonly cultivated blueberries [25]. Additionally, it tolerates high soil pH levels and has low soil fertility requirements, thriving in ordinary soils.

Given these characteristics, research on *V. duclouxii* is of great importance. Building on our prior work completing the first high-quality *T2T* genome sequence of *V. duclouxii* and studies on the mechanisms of anthocyanin biosynthesis, we conducted further research into the *V. duclouxii* GST gene family using bioinformatics and transcriptomic data. We identified 88 VdGSTs genes, divided into seven subfamilies, a notable expansion compared to other species like the *V. bracteatum*. The genes related to anthocyanin transport belong to the Phi subfamily. In the *Fragaria × ananassa*, GST genes associated with anthocyanins are predominantly clustered within the Phi class [33], suggesting that similar functions may be executed by the Phi subfamily GST genes in *V. duclouxii*. Notably, *V. duclouxii* not only exhibits a clear gene expansion but also shows significant clustering of GST genes on chromosomes 1, 2, 4, and 6. This gene clustering, largely corresponding with their subfamily classifications in the evolutionary tree, facilitates gene interactions and signaling, enhancing the organism’s adaptability to environmental changes [34]. The intense competition in *V. duclouxii*’s native habitat of the Ailao Mountains in Yunnan suggests that this gene clustering may be related to its environmental adaptability.

In terms of gene structure, although the 88 GST genes vary in length, they share a high homology in the conservative GST-N region, while the C-terminal is highly variable. This is similar to other plants where GSTs follow a basic two-domain fold pattern: an N-terminal domain and a C-terminal domain. Unlike the conservative N-terminal domain, the C-terminal domain varies significantly in sequence and topology, which leads to distinct hydrophobic substrate specificities in plant GSTs [35]. However, there is considerable variation in the number of introns and exons. This variability is a key factor in the diverse roles played by members of the *GST* gene family in plants, where the distribution of introns and exons and the number of introns are typical evolutionary markers of plant gene families [36]. In eukaryotes, introns are spliced by exons, and an increase in intron number enriches gene types and protein functions.

Gene duplication events are a significant evolutionary mechanism, providing potential avenues for changes in gene function and subsequent evolutionary developments [37]. The *Vaccinium* and *Rhododendron* genera diverged from a common ancestor between 43.6 and 57.9 million years ago (Mya). *Vaccinium* species such as *V. duclouxii* and *V. bracteatum* from China, alongside *V. darrowii* and *V. myrtillus* from North America and Europe, diverged between 10.7 and 20.4 Mya. Specifically, *V. duclouxii* and *V. bracteatum* diverged between 8.3 and 16.3 Mya [25]. An expansion of GST sequences with high content in *V. duclouxii* likely resulted from divergence from *V. bracteatum*, potentially due to intense competition with nearby species in Yunnan’s Ailao Mountains. Additionally, dispersed or tandem duplications also lead to an increase in gene count [38], with tandem duplication events in the *V. duclouxii* genome likely being a primary driver of gene family expansion. The GST genes, with a Ka/Ks ratio less than 0.5, indicate that these genes are highly conserved and have undergone purifying selection.

In the promoter regions of most GST genes, numerous cis-acting elements associated with hormones, stress responses, and photoreaction, as well as growth and development, were identified. This finding supports the potential role of GST genes in *V. duclouxii*’s response to various abiotic stresses. Research indicates that GST proteins participate in multiple biological processes in plants, such as growth, development, and resistance to biotic and abiotic stresses, and are widely distributed across plant tissues. They play a crucial role in herbicide tolerance and heavy metal stress detoxification [39]. Further research identified *VdGSTF11* during the fruiting stage, with transcriptomic analyses and qRT-PCR confirming its expression was significantly higher during the fruit color transition from green to red to black, showing co-expression with anthocyanin synthase in *V. duclouxii* [25]. Located on the plasma membrane, these results suggest that the *VdGSTF11* gene plays a critical role in anthocyanin synthesis and transport. The manipulation of GST genes, particularly those like *VdGSTF11* involved in anthocyanin transport, could lead to blueberry varieties with improved pigmentation, enhanced stress tolerance, and increased nutritional value. The use of advanced genomic tools, such as CRISPR/Cas9 and SNP chips, in the precise editing and selection of these genes could revolutionize the development of blueberries, optimizing them for both agronomic performance and market demand. This study provides a foundation for future gene cloning and transformation, functional validation, and the development of molecular markers for marker-assisted breeding.

## 4. Materials and Methods

### 4.1. Plant Materials

*V. duclouxii* is grown in the Flower Nursery Stock Base of Huazhong Agricultural University in Wuhan, China, for gene cloning. Mature leaves, flowers, and fruits at different ripening stages (immature fruits, partially ripe fruits, almost-ripe fruits, and fully mature fruits) from High Mountain Economic Plant Research Institute in Lijiang City, Yunnan Province, China, were harvested as described [23].

### 4.2. Identification and Phylogenetic Analysis of VdGST Gene Family in V. duclouxii

The genomic and annotation data for *V. duclouxii* were sourced from the project group’s previous research sequencing efforts recorded in NGDC [25]. GST protein sequences of *A. thaliana* (52 GSTs) were downloaded from TAIR (https://www.arabidopsis.org/ accessed on 10 May 2023). Hidden Markov model sequences for GSTs, identified as PF00043 (GST_C) and PF02798 (GST_N), were obtained from the Pfam database (http://pfam.xfam.org accessed on 10 May 2023). We utilized HMMER 3.0 software to identify the GST gene family in *V. duclouxii*. Candidate genes were verified for structural domains using the Conserved Domain Database (CDD) (https://www.ncbi.nlm.nih.gov/Structure/cdd/wrpsb.cgi accessed on 12 May 2023). To characterize the physicochemical properties of the identified VdGSTs, we analyzed amino acid composition, sequence lengths, molecular weights, isoelectric points (pI), instability indices, hydropathy (GRAVY index), and aliphatic indices using the ExPASy-ProParam tool (https://web.ExPASy.org/protparam/ accessed on 15 May 2023). Subcellular localization predictions for all members were performed using the Cell-PLoc 2.0 website (http://www.csbio.sjtu.edu.cn/bioinf/plant-multi/ accessed on 5 December 2023) and the CELLO v2.5 subcellular localization predictor (http://cello.life.nctu.edu.tw/ accessed on 5 December 2023).

A total of 88 *VdGSTs* from *V. duclouxii*, 52 *VbGSTs* from *V. bracteatum*, and 52 *AtGSTs* from *A. thaliana* were conducted by aligning using the default settings of MAFFT v7.158 [40]. The evolutionary tree was pruned and aligned using the trimAL program. The phylogenetic tree was constructed using the maximum likelihood method (ML) with the iqtree2 software [41], setting the bootstrap value at 1000. The tree was visualized and refined using the iTOLv5 [42].

### 4.3. Chromosomal Localization, Gene Structure Prediction, and Motif Composition of VdGSTs

Based on the genomic annotation data of *V. duclouxii*, intron–exon information was extracted to construct gene structure diagrams. The MEME Suite (http://meme-suite.org/tools/meme accessed on 6 December 2023) was employed for protein motif analysis. The chromosomal positions of all GST genes in *V. duclouxii* were determined, and their locations on the chromosomes were visualized using the MapInspect tool in TBtools, combined with genomic structure annotation files. Gene cluster positions were further visualized using the online platform ChiPlot (https://www.chiplot.online/ accessed on 8 December 2023).

### 4.4. Comparative Genomics Analysis of V. duclouxii with Four Other Species

*A. thaliana* genome data were sourced from TAIR. *R. henanense* genome data were downloaded from the NGDC (https://ngdc.cncb.ac.cn/gwh/Assembly/22219/show accessed on 8 December 2023) [43], and *V. bracteatum* from NGDC [44]. The genome data for *V. darrowii* were obtained from NCBI [45]. The MCScanX tool was used to analyze the syntenic regions of the *VdGSTs* genes. Comparative synteny analysis was performed between them. Based on these analyses, the Simple Ka/Ks Calculator module in TBtools software v2.086 was utilized to calculate Ka and Ks substitution rates for each gene pair. The environmental selective pressures on each gene pair were assessed using the Ka/Ks ratio, and results were visualized using the ggplot2 v3.5.1 package in R [46].

### 4.5. Prediction of Cis-Regulatory Elements in VdGSTs Gene Promoters

The 2.0 kb upstream DNA sequences of the *VdGSTs* genes were submitted to the PlantCARE database (https://bioinformatics.psb.ugent.be/webtools/plantcare/html/ accessed on 10 December 2023) for the prediction of cis-regulatory elements [47]. These promoters cis-acting regulatory elements were analyzed and visualized using TBtools.

### 4.6. Expression Analysis of VdGSTs Genes Based on RNA-Seq Data

To explore the fundamental expression patterns of *VdGSTs* genes during fruit development and elucidate their significance in this process, specimens of *V. duclouxii* fresh leaves (L), flowers (F), and fruits at different maturity stages (F1 green fruit, F2 half-red fruit, F3 red fruit, and F4 fully black fruit) were collected. All *VdGSTs* genes across different tissues were screened for their FPKM values, discarding transcripts with average FPKM values of less than 1. A heatmap of gene expression was generated using TBtools software.

### 4.7. Validation of Transcriptome Data with qRT-PCR

To confirm the accuracy of RNA-Seq data, qRT-PCR was conducted. RNA samples were reverse-transcribed using a reverse transcription kit (Toyobo, Osaka, Japan), with specific primers designed (Table S3), and verified against the local transcriptome library using TBtools. The qRT-PCR was performed using the SYBR PreMix Ex Taq Kit (Takara, Kusatsu, Japan) on a Roche LightCycler 96 System (Roche, Basel, Switzerland). The qRT-PCR program followed the parameters described [48]. The expression levels were calculated using the 2^−ΔΔC^_T_ method [49], with GADPH as the internal control gene [50]. Each sample was analyzed with three biological replicates and two technical replicates.

### 4.8. Subcellular Localization Analysis of VdGSTF11

After double digestion of the pSuper1300-GFP vector with *S*alI and *K*pnI restriction endonucleases, the coding region of *VdGSTF11* (excluding the stop codon) was cloned into the pSuper1300-GFP vector using the ClonExpress^®^II One Step Cloning Kit (Vazyme, Nanjing, China). The resulting pSuper1300-*VdGSTF11-GFP* vector was transformed into the *Agrobacterium tumefaciens* strain GV3101. Following transformation, the culture was plated on kanamycin-resistant plates and incubated at 28 °C for 2 days in an inverted incubator. Colonies were verified via PCR and sequencing to identify positive strains, which were subsequently cultured in 50 mL of YEB liquid medium (containing 50 mg/L kanamycin, 10 mM MES, and 100 µM acetosyringone) at 28 °C and 200 rpm until the OD600 reached 1.0–1.5. The culture was then centrifuged at 5000 rpm for 5 min, the supernatant discarded, and the pellet resuspended in a suspension solution (10 mM MES, 10 mM magnesium chloride, 100 µM acetosyringone, pH 5.7) to an approximate OD600 of 0.8. The suspension was mixed well, settled at room temperature in the dark for 3 h, and used for *Nicotiana benthamiana* leaf infiltration.

Healthy tobacco leaves were selected, and 1 mL of the bacterial suspension was injected into the underside of the leaves using a sterile syringe without a needle. After injection, the tobacco was incubated in the dark for 60 h. GFP fluorescence was examined using a confocal laser scanning microscope (Leica Microsystems TCS-SP8, Wetzlar, Germany).

## 5. Conclusions

This study comprehensively identified and analyzed the GST gene family in *V. duclouxii*. It systematically examined the gene structures, evolutionary relationships, duplication events, and cis-acting elements in the promoters of the GST gene family. A total of 88 GST genes were identified, characterized by conserved N-terminal regions and variable C-terminal regions. By constructing a phylogenetic tree, seven subfamilies were delineated. Members within each subfamily are highly conserved while exhibiting distinct sequence and structural features across different subfamilies, each maintaining a similar number of gene structures. These 88 GST genes are distributed across 12 chromosomes in *V. duclouxii*, with some forming gene clusters predominantly influenced by purifying selection during evolutionary processes. Promoter analysis for each gene member revealed a predominance of various hormone response elements and stress response elements. Transcriptomic and qRT-PCR analyses indicated that *VdGSTF11* is expressed on the plasma membrane and shows significantly higher expression during the fruit’s color transition from green to red to black, suggesting co-expression with *V. duclouxii*’s anthocyanin synthase. This indicates a crucial role for the *VdGSTF11* gene in anthocyanin accumulation. Our research provides a theoretical basis for further exploration into the molecular mechanisms regulating anthocyanin accumulation in *V. duclouxii*, guiding molecular breeding efforts and offering valuable resources for improving hybrid breeding programs. This is essential for developing enhanced anthocyanin-rich blueberry varieties.

## Data Availability

Data are contained within the article and Supplementary Materials.

## Supplementary Materials

The following supporting information can be downloaded at: https://www.mdpi.com/article/10.3390/plants13111497/s1, Table S1: Prediction of physicochemical properties of the *VdGSTs*; Table S2: Duplication type of *VdGSTs*; Table S3: Primers used in this study.
