# Supplementary material for: Genome-Wide Identification Analysis of GST Gene Family in Wild Blueberry Vaccinium duclouxii and Their Impact on Anthocyanin Accumulation
Source: Plants (Basel). 2024 May 29;13(11):1497. doi: 10.3390/plants13111497 (PMC11174658; doi:10.3390/plants13111497)
Supplement: Supplementary file 1 [file plants-13-01497-s001.zip › plants-3018963-supplementary.pdf]

Supplemental information

Supplemental Table S1. Prediction of physicochemical properties of the *VdGSTs*

gene name	Sequence ID	Category	Number of Amino Acid	Molecular Weight	Theoretical pI	Instability Index	Aliphatic Index	Grand Average of Hydropathicity	Predicted subcellular location
VdDHAR1	VducChr10G329370	DHAR	122	13459.75	4.98	20.94	108.69	0.367	Cytoplasm
VdDHAR2	VducChr10G329430	DHAR	127	14578.03	5.01	22.31	88.35	0.091	Cytoplasm
VdDHAR3	VducChr10G351570	DHAR	225	25018.07	5.61	28.17	100.58	-0.003	Cytoplasm
VdDHAR4	VducChr11G239680	DHAR	238	26380.53	5.17	28.39	94.29	-0.02	Cytoplasm
VdDHAR5	VducChr4G216350	DHAR	95	10892.72	5.12	40.83	85.16	0.036	Cytoplasm\Nucleus
VdGSTF1	VducChr2G020240	Phi	217	24870.92	8.78	35.34	84.42	-0.242	Cytoplasm
VdGSTF2	VducChr5G042060	Phi	330	38313.68	8.79	41.14	96.27	-0.248	Cytoplasm
VdGSTF3	VducChr6G148500	Phi	216	24476.92	6.92	42.81	82.64	-0.461	Cytoplasm
VdGSTF4	VducChr6G148510	Phi	237	27605.14	6.93	42.84	90.42	-0.284	Cytoplasm
VdGSTF5	VducChr6G148520	Phi	230	25821.11	5.83	43.05	98.87	-0.047	Cytoplasm
VdGSTF6	VducChr6G148530	Phi	149	16940.33	6.59	43.99	76.51	-0.47	Cytoplasm
VdGSTF7	VducChr6G148540.	Phi	232	27362.99	6.38	44.02	89.91	-0.249	Cytoplasm
VdGSTF8	VducChr6G148550	Phi	221	25514.75	5.87	44.55	97.92	-0.204	Cytoplasm
VdGSTF9	VducChr6G148560	Phi	222	25119.54	5.38	44.6	101.4	-0.01	Cytoplasm
VdGSTF10	VducChr6G148590	Phi	232	27423.06	6.17	45.1	92.84	-0.266	Cytoplasm
VdGSTF11	VducChr8G260070	Phi	264	29975.38	8.89	47.33	87.92	-0.263	Cytoplasm
VdGSTL1	VducChr11G240770	Lambde	223	24733.63	5.43	28.5	97.13	-0.032	Nucleus
VdGSTL2	VducChr6G139280	Lambde	222	25026.42	5.77	42.41	104.01	-0.039	Chloroplast
VdGSTL3	VducChr9G296900	Lambde	69	7860.86	4.38	49.87	67.97	-0.258	Cytoplasm

VdGSTL4	VducChr9G296910	Lambde	257	29256.71	6.13	50.94	91.48	-0.222	Cytoplasm
VdGSTT1	VducChr10G351050	Theta	212	23929.65	5.43	28.03	95.14	-0.282	Cytoplasm
VdGSTT2	VducChr10G351070	Theta	212	23941.66	5.57	28.03	94.2	-0.33	Cytoplasm
VdGSTT3	VducChr6G132750	Theta	232	27422.01	5.35	42.06	93.66	-0.181	Chloroplast
VdGSTT4	VducChr7G392220	Theta	551	62511.64	5.64	45.22	90.44	-0.387	Chloroplast
VdGSTT5	VducChr8G274150	Theta	265	31398.46	9.23	48.16	89.32	-0.326	Chloroplast
VdGSTU1	VducChr10G346110	Tau	213	23636.41	5.57	22.91	101.74	0.067	Cytoplasm
VdGSTU2	VducChr10G346140	Tau	233	26281.51	5.72	23.89	97.85	-0.179	Nucleus
VdGSTU3	VducChr10G350130	Tau	212	23789.49	7.06	26.98	87.03	-0.132	Cytoplasm
VdGSTU4	VducChr10G350140	Tau	226	25064.15	6.35	27.29	90.31	-0.108	Cytoplasm
VdGSTU5	VducChr12G362870	Tau	223	25040.22	5.67	28.88	101.35	0.002	Cytoplasm
VdGSTU6	VducChr12G377770	Tau	228	25230.35	5.58	28.98	105.61	0.018	Cytoplasm
VdGSTU7	VducChr12G381840	Tau	226	24868.77	5.73	29.17	89.38	-0.083	Cytoplasm
VdGSTU8	VducChr1G160330	Tau	159	17798.53	5.31	29.74	84.78	-0.211	Chloroplast\Cytoplasm
VdGSTU9	VducChr1G160340	Tau	215	24346	5.99	30.25	83.91	-0.22	Cytoplasm
VdGSTU10	VducChr1G160350	Tau	219	25756.53	5.3	31.33	84.98	-0.395	Cytoplasm
VdGSTU11	VducChr1G160360	Tau	222	24851.77	6.08	32.67	97.66	-0.063	Cytoplasm
VdGSTU12	VducChr1G160370	Tau	227	24996.06	5.57	32.89	86.48	-0.019	Cytoplasm
VdGSTU13	VducChr1G160390	Tau	718	81295.27	8.28	32.93	86.48	-0.354	Cytoplasm
VdGSTU14	VducChr1G160440	Tau	231	25652.74	5.87	33.04	91.69	-0.11	Cytoplasm
VdGSTU15	VducChr1G160450	Tau	222	25542.78	5.66	33.23	94.46	-0.141	Cytoplasm
VdGSTU16	VducChr1G160470	Tau	214	24076.96	6.07	33.43	87.94	-0.052	Cytoplasm
VdGSTU17	VducChr1G180760	Tau	212	23606.3	5.6	33.57	99.81	0.077	Cytoplasm
VdGSTU18	VducChr1G180770	Tau	218	25120.01	5.77	33.85	83.58	-0.322	Nucleus
VdGSTU19	VducChr1G180780	Tau	325	36969.88	5.95	34.76	74.22	-0.44	Cytoplasm
VdGSTU20	VducChr2G015650	Tau	343	38659.43	5.19	34.83	87.26	-0.065	Cytoplasm

VdGSTU21	VducChr2G017150	Tau	420	47996.19	5.86	35.11	72.24	-0.468	Chloroplast\Cytoplasm
VdGSTU22	VducChr2G024230	Tau	216	24178.97	5.96	36.38	89.91	0.013	Cytoplasm
VdGSTU23	VducChr2G024250	Tau	211	23272.89	4.67	36.82	61.94	-0.699	Cytoplasm
VdGSTU24	VducChr2G024260	Tau	218	24921	5.7	36.85	99.31	-0.173	Chloroplast\Cytoplasm
VdGSTU25	VducChr2G024270	Tau	240	27279.79	6.03	36.88	94.29	-0.252	Cytoplasm
VdGSTU26	VducChr2G024290	Tau	461	51992.44	5.41	37.37	78.5	-0.411	Cytoplasm
VdGSTU27	VducChr2G024300	Tau	166	18568.54	8.56	37.41	94.52	-0.039	Cytoplasm
VdGSTU28	VducChr2G024320	Tau	421	48262.71	5.94	37.43	79.22	-0.395	Nucleus
VdGSTU29	VducChr2G024330	Tau	216	24529.51	5.49	37.65	91.16	-0.18	Cytoplasm
VdGSTU30	VducChr2G026290	Tau	213	24509.45	5.47	37.7	101.6	-0.121	Cytoplasm
VdGSTU31	VducChr2G026300	Tau	219	25567.4	5.75	37.8	83.24	-0.413	Cytoplasm
VdGSTU32	VducChr2G026310	Tau	265	29475.15	8.67	37.95	86.42	-0.254	Cytoplasm
VdGSTU33	VducChr2G026390	Tau	232	26231.58	5.42	38.41	99.18	-0.164	Cytoplasm
VdGSTU34	VducChr2G026400	Tau	212	23592.36	5.99	38.46	93.73	-0.235	Cytoplasm
VdGSTU35	VducChr2G028810	Tau	160	17453.19	6.5	38.54	90.87	0.061	Cytoplasm
VdGSTU36	VducChr2G028820	Tau	220	25351.33	5.28	38.91	91.32	-0.229	Cytoplasm
VdGSTU37	VducChr2G028830	Tau	94	11023.4	4.9	38.96	64.47	-0.644	Cytoplasm
VdGSTU38	VducChr3G100110	Tau	178	19949.93	8.4	39.46	87.13	-0.195	Cytoplasm
VdGSTU39	VducChr3G104210	Tau	472	54204.98	5.6	39.52	104.43	-0.156	Cytoplasm
VdGSTU40	VducChr4G194720	Tau	221	25544.61	6.96	39.69	84.21	-0.377	Cytoplasm
VdGSTU41	VducChr4G194730	Tau	232	27236.7	5.82	39.81	95.34	-0.295	Cytoplasm
VdGSTU42	VducChr4G194740	Tau	442	50602.6	5.52	40.39	93.28	-0.203	Cytoplasm
VdGSTU43	VducChr4G194750	Tau	223	25382.18	6.11	40.46	95.38	-0.244	Cytoplasm
VdGSTU44	VducChr4G194760	Tau	253	29094.84	5.56	40.52	100.91	-0.115	Cytoplasm
VdGSTU45	VducChr5G047280	Tau	222	25706.54	5.45	41.2	89.05	-0.199	Cytoplasm
VdGSTU46	VducChr5G047290	Tau	157	17779.62	5.21	41.64	83.31	-0.249	Cytoplasm

VdGSTU47	VducChr5G047310	Tau	423	48335.63	5.72	41.68	75.39	-0.498	Cytoplasm
VdGSTU48	VducChr6G136190	Tau	231	27253.9	6.86	42.19	93.2	-0.242	Cytoplasm
VdGSTU49	VducChr8G282030	Tau	223	24928.24	5.17	48.62	82.33	-0.496	Cytoplasm
VdGSTU50	VducChr8G282040	Tau	237	27553.39	5.24	48.97	79.83	-0.52	Cytoplasm
VdGSTU51	VducChr8G282050	Tau	231	25855.39	5.34	48.98	79.52	-0.452	Cytoplasm
VdGSTU52	VducChr9G305380	Tau	303	34771.18	6.11	53.92	94.59	-0.093	Cytoplasm
VdGSTU53	VducChr9G294510	Tau	211	24503.17	5.91	49.15	92.32	-0.271	Cytoplasm
VdGSTU54	VducChr9G294520	Tau	330	37525.78	9.22	49.28	76.88	-0.43	Cytoplasm
VdGSTU55	VducChr9G294530	Tau	174	20063.15	7.61	49.5	77.24	-0.489	Cytoplasm
VdGSTZ1	VducChr12G360050	Zeta	228	25498.73	5.85	28.86	103.9	-0.007	Cytoplasm
VdGSTZ2	VducChr2G031940	Zeta	214	25133.29	5.73	39.19	90.14	-0.259	Cytoplasm
VdGSTZ3	VducChr9G302820	Zeta	237	27374.15	4.91	52.71	80.59	-0.465	Cytoplasm
VdTCHQD1	VducChr2G019130	TCHQD	136	15650.48	8.65	35.2	107.5	-0.093	Cytoplasm
VdTCHQD2	VducChr2G032790	TCHQD	228	26436.88	5.48	39.28	107.63	-0.196	Cytoplasm
VdTCHQD3	VducChr7G411180	TCHQD	205	23506.17	5.19	46.35	106.49	0.206	Cytoplasm
VdTCHQD4	VducChr9G301800	TCHQD	237	27090.38	6.8	52.01	92.62	-0.27	Cytoplasm
VdTCHQD5	VducChr9G301860	TCHQD	129	14788.84	5.25	52.21	72.56	-0.702	Cytoplasm

Table S2 Duplication type of *VdGSTs*

Gene Name	Duplication type	Gene Name	Duplication type
VducChr2G015650.1	Dispersed	VducChr2G024330.1	Tandem
VducChr2G017150.1	Dispersed	VducChr2G026290.1	Tandem
VducChr2G019130.1	Dispersed	VducChr2G026300.1	Tandem
VducChr2G020240.1	Dispersed	VducChr2G026310.1	Tandem
VducChr2G031940.1	Dispersed	VducChr2G026390.1	Tandem
VducChr3G104210.1	Dispersed	VducChr2G026400.1	Tandem
VducChr4G216350.1	Dispersed	VducChr2G028810.1	Tandem
VducChr5G042060.1	Dispersed	VducChr2G028820.1	Tandem
VducChr6G139280.1	Dispersed	VducChr2G028830.1	Tandem
VducChr7G392220.1	Dispersed	VducChr4G194730.1	Tandem
VducChr8G260070.1	Dispersed	VducChr4G194740.1	Tandem
VducChr8G274150.1	Dispersed	VducChr4G194750.1	Tandem
VducChr9G302820.1	Dispersed	VducChr4G194760.1	Tandem
VducChr9G305380.1	Dispersed	VducChr5G047280.1	Tandem
VducChr10G351570.1	Dispersed	VducChr5G047290.1	Tandem
VducChr11G240770.1	Dispersed	VducChr6G148500.1	Tandem
VducChr12G360050.1	Dispersed	VducChr6G148510.1	Tandem
VducChr12G362870.1	Dispersed	VducChr6G148520.1	Tandem
VducChr12G381840.1	Dispersed	VducChr6G148530.1	Tandem
VducChr1G160390.1	Proximal	VducChr6G148540.1	Tandem
VducChr1G160470.1	Proximal	VducChr6G148550.1	Tandem
VducChr2G024290.1	Proximal	VducChr6G148560.1	Tandem
VducChr2G024300.1	Proximal	VducChr8G282030.1	Tandem

VducChr5G047310.1	Proximal	VducChr8G282040.1	Tandem
VducChr6G132750.1	Proximal	VducChr8G282050.1	Tandem
VducChr6G148590.1	Proximal	VducChr9G294510.1	Tandem
VducChr9G301860.1	Proximal	VducChr9G294520.1	Tandem
VducChr10G329430.1	Proximal	VducChr9G294530.1	Tandem
VducChr10G346110.1	Proximal	VducChr9G296900.1	Tandem
VducChr10G351070.1	Proximal	VducChr9G296910.1	Tandem
VducChr7G411180.1	Singleton	VducChr10G346140.1	Tandem
VducChr1G160330.1	Tandem	VducChr10G350130.1	Tandem
VducChr1G160340.1	Tandem	VducChr10G350140.1	Tandem
VducChr1G160350.1	Tandem	VducChr1G180760.1	WGD or Segmental
VducChr1G160360.1	Tandem	VducChr2G024230.1	WGD or Segmental
VducChr1G160370.1	Tandem	VducChr2G032790.1	WGD or Segmental
VducChr1G160440.1	Tandem	VducChr3G100110.1	WGD or Segmental
VducChr1G160450.1	Tandem	VducChr4G194720.1	WGD or Segmental
VducChr1G180770.1	Tandem	VducChr6G136190.1	WGD or Segmental
VducChr1G180780.1	Tandem	VducChr9G301800.1	WGD or Segmental
VducChr2G024250.1	Tandem	VducChr10G329370.1	WGD or Segmental
VducChr2G024260.1	Tandem	VducChr10G351050.1	WGD or Segmental
VducChr2G024270.1	Tandem	VducChr11G239680.1	WGD or Segmental
VducChr2G024320.1	Tandem	VducChr12G377770.1	WGD or Segmental

Supplemental Table S3. Primers used in this study

ID	Primer name	Primer sequences(5' to 3')
Realtime PCR	VdGSTL4_RT_F	TTCTGAAGAGTTGTTGGCGTAC
Realtime PCR	VdGSTL4_RT_R	GCAATGTCCACCAGACTGAATT
Realtime PCR	VdGSTU13_RT_F	ATGGCAAGCAAGGAAGTAGTAA
Realtime PCR	VdGSTU13_RT_R	AATCAACACTGGCACCTTCTT
Realtime PCR	VdGSTU22_RT_F	CCTCGTCATTGTCCAGTATGTT
Realtime PCR	VdGSTU22_RT_R	CCTTCTTGGCTTCCTCTGTTG
Realtime PCR	VdGSTZ2_RT_F	CTTCCGTAGTTCTTGCTCTTACC
Realtime PCR	VdGSTZ2_RT_R	AGTACAATGTCCTCATCCACCAA
Realtime PCR	VdGSTF11_RT_F	GACAAGTTCCTGCCATAGAAGAT
Realtime PCR	VdGSTF11_RT_R	AGTTGAAGGACCAAGTTGTAGAC
Realtime PCR	VdGADPH_RT_F	TCGATTACTGCCACTCAGAAG
Realtime PCR	VdGADPH_RT_R	TGCTGGCAGAACTTTTCCTAC
linking to pSuper1300GFP	VdGSTF11-pS_F	TGCAGGGGCCCGGGGTCGACATGGTGGTTAAAGTTTATGGTCC
linking to pSuper1300GFP	VdGSTF11-pS_R	CCCTTGCTCACCATGGTACCATCCATAAGCTTCATTATTTTCTTC
